# Insights into the Role of LncRNAs and miRNAs in Glioma Progression and Their Potential as Novel Therapeutic Targets

**DOI:** 10.3390/cancers15133298

**Published:** 2023-06-22

**Authors:** Mateusz Kciuk, Esam Bashir Yahya, Montaha Mohamed Ibrahim Mohamed, Muhanad A. Abdulsamad, Abdulmutalib A. Allaq, Adrianna Gielecińska, Renata Kontek

**Affiliations:** 1 Department of Molecular Biotechnology and Genetics, University of Lodz, 90-237 Lodz, Poland; 2 Doctoral School of Exact and Natural Sciences, University of Lodz, 90-237 Lodz, Poland; 3 Bioprocess Technology Division, School of Industrial Technology, Universiti Sains Malaysia, Penang 11800, Malaysia; 4 Faculty of Applied Medical Sciences, Mohail Aseer, King Khalid University, Abha 62529, Saudi Arabia; 5 Department of Molecular Biology, Faculty of Science, Sabratha University, Sabratha 00218, Libya; 6 Faculty of Applied Science, Universiti Teknologi MARA, Shah Alam 40450, Malaysia

**Keywords:** glioma, long RMA, micro non-coding RNAs, gene therapy, brain tumors

## Abstract

**Simple Summary:**

Recent research has revealed the involvement of long non-coding RNAs (lncRNAs) and micro RNAs (miRNAs) in glioma development and advancement, suggesting their potential as biomarkers and therapeutic targets. Late-stage diagnosis and the limited effectiveness of conventional treatments contribute to poor glioma outcomes, highlighting the need for early detection methods. Advances in biotechnology and neuroscience have shed light on non-coding RNAs role in glioma angiogenesis and progression. This review explores the impact of lncRNAs and miRNAs on gene regulation, either promoting or inhibiting glioma progression and establishes connections between these crucial cancer-related molecules.

**Abstract:**

Accumulating evidence supports that both long non-coding and micro RNAs (lncRNAs and miRNAs) are implicated in glioma tumorigenesis and progression. Poor outcome of gliomas has been linked to late-stage diagnosis and mostly ineffectiveness of conventional treatment due to low knowledge about the early stage of gliomas, which are not possible to observe with conventional diagnostic approaches. The past few years witnessed a revolutionary advance in biotechnology and neuroscience with the understanding of tumor-related molecules, including non-coding RNAs that are involved in the angiogenesis and progression of glioma cells and thus are used as prognostic biomarkers as well as novel therapeutic targets. The emerging research on lncRNAs and miRNAs highlights their crucial role in glioma progression, offering new insights into the disease. These non-coding RNAs hold significant potential as novel therapeutic targets, paving the way for innovative treatment approaches against glioma. This review encompasses a comprehensive discussion about the role of lncRNAs and miRNAs in gene regulation that is responsible for the promotion or the inhibition of glioma progression and collects the existing links between these key cancer-related molecules.

## 1. Introduction

The majority of the human genome is composed of non-protein coding genes. These genes do not code for proteins but instead produce various types of RNA molecules that are not translated into proteins [1]. For a long time, the non-translated regions of the human genome were often referred to as “junk DNA” because their function was not well understood. However, in the last two decades, advancements in genomic research have revealed that these non-coding regions play critical roles in gene regulation and other important biological processes [2]. In general, non-coding RNAs can be classified into small RNAs, which include families such as small interfering RNA (siRNA), microRNA (miRNA), piwi interacting RNA (piRNA), as well as the long non-coding RNA (lncRNA) [3]. Calin et al. [4] investigated the involvement of non-coding RNAs in cancer development for the first time in 2002. Since then, non-coding RNAs have been the subject of extensive research in the field of cancer biology. Their roles in cancer development and progression have been investigated in numerous studies. These studies have explored various aspects of non-coding RNAs, including their mechanisms of action, genomic characteristics, biogenesis, functional phenotypes in experimental models, and involvement in cellular pathways [5]. Gliomas are a type of brain tumor that arise from glial cells in the central nervous system. The classification of gliomas traditionally relied on histological features, but advancements in molecular biology have led to a more comprehensive classification system [6]. While gliomas can occur in both adults and children, there are some notable differences between gliomas in adults and pediatrics in terms of their characteristics, clinical presentation, and treatment approaches. Adult gliomas often exhibit genetic alterations such as mutations in the IDH1/2 genes, EGFR amplification, and loss of chromosome 10q. Pediatric gliomas, on the other hand, are frequently associated with genetic alterations such as BRAF fusion or mutation, KIAA1549-BRAF fusion, and mutations in H3F3A or HIST1H3B [7]. The latest World Health Organization (WHO) classification of gliomas, as of 2021, is known as the CNS5 classification, which classifies the glioma into several major groups, as presented in Table 1 [8]. It is important to note that this is a simplified overview, and there are additional subtypes and variations within each category. Furthermore, the CNS5 classification incorporates molecular markers such as IDH mutation status, 1p/19q codeletion, and MGMT promoter methylation status to provide a more precise classification of gliomas [9,10]. p53 mutations are relatively common in high-grade gliomas. It is estimated that p53 mutations occur in approximately 30% to 40% of glioblastoma cases. However, the frequency of p53 mutations can vary across different glioma subtypes and grades [11,12]. The presence of p53 mutations in gliomas has been associated with poorer prognosis and shorter overall survival. Patients with p53-mutated gliomas tend to have more aggressive tumor behavior, increased resistance to therapy, and a higher likelihood of disease recurrence.

**Figure 1:**
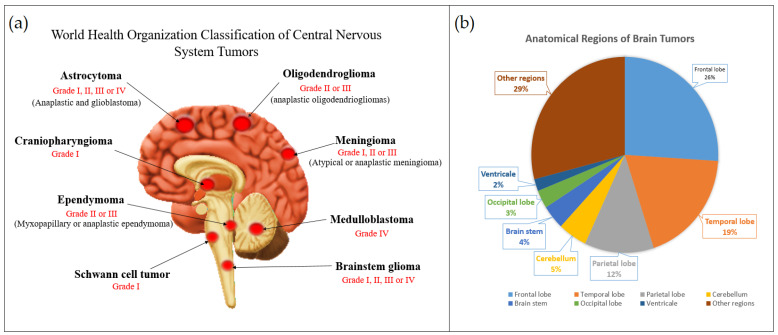
Illustration of the most common central nervous system tumors; (**a**) WHO classification of each type, (**b**) the percentage of CNS tumors based on the anatomical region.

The classification of gliomas is essential for guiding treatment decisions, predicting prognosis, and conducting research to develop targeted therapies tailored to specific subtypes of glioma. Malignant gliomas are the most common primary brain tumors in adults. The annual incidence rate varies across populations, but it is estimated to be around 5–10 cases per 100,000 people [13,14]. The prognosis for malignant gliomas is generally poor. The survival rates can vary depending on various factors, including the specific subtype of glioma, the age of the patient, the extent of surgical resection, and molecular characteristics. However, in general, the median overall survival for glioblastoma, the most common malignant glioma, is typically around 12–15 months [14,15]. Malignant gliomas have a high likelihood of recurrence even after initial treatment. The majority of patients experience tumor regrowth within a year after diagnosis, despite undergoing surgery, radiation therapy, and chemotherapy. Ongoing research and clinical trials aim to improve the outcomes for patients with malignant gliomas. These efforts focus on developing novel treatment strategies, including targeted therapies, immunotherapies, and gene therapies, to enhance survival rates and quality of life. The distribution of glioma types differs between adults and pediatrics. In adults, the most common type of glioma is glioblastoma multiforme, which is an aggressive and high-grade tumor. In pediatrics, the most common glioma is pilocytic astrocytoma, which is a low-grade tumor. Other common glioma types in children include diffuse intrinsic pontine glioma and medulloblastoma. In adults, gliomas are commonly found in the cerebral hemispheres, particularly in the frontal and temporal lobes. Pediatric gliomas often occur in the posterior fossa, which includes the cerebellum and brainstem [16]. Figure 1 presents the most common CNS tumors and the recent World Health Organization (WHO) classification of each type, together with the percentage of CNS tumors based on the anatomical region [17,18].

Despite advances in the diagnosis and treatment of glioma, the median survival time after diagnosis of high-grade glioma (such as glioblastoma) remains low, approximately 15 months, and with a 5-year overall survival rate of only 6.8% [19,20]. In recent decades, significant progress has been made in understanding the biology of cancer cells and the characteristics of tumors, including gliomas. Research in the field of glioma has focused on identifying alterations in gene expression, gene mutations, and epigenetic modifications [21]. Epigenetic modifications contribute to the molecular subtyping of gliomas. For example, DNA methylation patterns can classify gliomas into distinct subgroups, such as the CpG island methylator phenotype (G-CIMP) subgroup, which is associated with a better prognosis. These subtypes have different genetic and epigenetic profiles, reflecting diverse underlying biology and potential therapeutic responses [22]. Epigenetic alterations contribute to the heterogeneity observed within gliomas. Different regions within the same tumor may display distinct epigenetic patterns, resulting in intratumoral heterogeneity. This heterogeneity affects various aspects, including the response to therapy and the emergence of drug-resistant subclones. Epigenetic alterations play a critical role in therapy resistance, including resistance to alkylating agents such as temozolomide (TMZ), which is commonly used in glioma treatment. DNA methylation changes can affect the expression of genes involved in DNA repair pathways, leading to decreased sensitivity to alkylating agents. In gliomas, promoter methylation of the DNA repair gene O^6-methylguanine-DNA methyltransferase (MGMT) is associated with increased TMZ responsiveness [23]. Epigenetic alterations can serve as prognostic and diagnostic biomarkers in gliomas. Abnormal expression of these factors during glioma angiogenesis has led scientists to explore their potential as biomarkers for diagnosing malignancy levels and assessing the therapeutic effectiveness of anticancer drugs. Manipulating specific RNAs through knockdown or over-expression has shown promise in inhibiting glioma growth. The complex interactions between these molecules in gene expression regulation have provided researchers with valuable insights into the roles of individual molecules in glioma development and their mechanisms of action. Each year, a significant number of publications are released, often highlighting the potential of these molecules in glioma diagnosis and therapy [24,25,26]. The present review delivers a comprehensive discussion about the role of long and micro non-coding RNAs in glioma angiogenesis, diagnosis, and therapy.

## 2. LncRNAs in Gliomas

Glioma progression refers to the development and growth of glioma tumors in the brain. Glioma progression is a complex and multifactorial process involving various genetic, molecular, and cellular changes. The prognosis and survival rates for gliomas differ between adults and pediatrics. Overall, pediatric gliomas tend to have better prognoses compared to gliomas in adults. This is partly due to the prevalence of low-grade tumors in children, which generally have better outcomes than high-grade tumors such as glioblastoma. However, certain pediatric gliomas, such as DIPG, have particularly poor prognoses [27]. The mechanism of glioma progression involves a complex interplay of genetic, molecular, cellular, and microenvironmental factors [28]. Generally, glioma progression is driven by the accumulation of genetic mutations. In adults, mutations in genes such as isocitrate dehydrogenase 1 and 2 (IDH1/2), epidermal growth factor receptor (EGFR), tumor protein p53 (TP53), and phosphatase and tensin homolog (PTEN) are frequently observed. In pediatric gliomas, specific genetic alterations are more commonly seen, such as BRAF fusion or mutation, KIAA1549-BRAF fusion, and mutations in histone genes (H3F3A or HIST1H3B). These mutations can disrupt normal cell growth control mechanisms and promote uncontrolled proliferation [29]. Various genes are commonly altered in gliomas, including TP53, IDH1/2, EGFR, PTEN, CDKN2A, and others. These mutations can disrupt critical signaling pathways involved in cell proliferation, survival, invasion, and angiogenesis [30,31]. The tumor microenvironment plays a crucial role in glioma progression. Gliomas interact with surrounding cells, including immune cells, stromal cells, and endothelial cells, which can promote tumor growth and invasion [31]. The microenvironmental factors, such as cytokines, growth factors, and extracellular matrix components, can modulate glioma cell behavior and facilitate progression. Gliomas exhibit invasive behavior, infiltrating adjacent brain tissues. The process of infiltration involves the migration of glioma cells into the surrounding normal brain parenchyma. Glioma cells can invade along white matter tracts and blood vessels, making complete surgical removal challenging [32]. Gliomas induce the formation of new blood vessels, a process known as angiogenesis, to ensure a blood supply for their growth. Glioma cells release pro-angiogenic factors, including vascular endothelial growth factor (VEGF), to stimulate the proliferation and migration of endothelial cells. This results in the formation of an abnormal network of blood vessels within the tumor. Rapid cellular proliferation of adult gliomas during tumor growth leads to increased oxygen demand by the tumor cells. This increased demand is a result of heightened cellular metabolism associated with the process of cell division. As a consequence, localized hypoxia can occur within the tumor microenvironment. The presence of hypoxia can have various effects on tumor biology, including influencing tumor aggressiveness and treatment response. [33]. Recent investigations revealed that hypoxia significantly influences additional aspects of tumor angiogenesis, such as vessel patterning, maturation, and even function [34,35]. In response to hypoxia, various pro-angiogenic pathways can be up-regulated. These pathways play a crucial role in promoting vessel growth and mediating key aspects of endothelial cell biology, vascular support, and stromal cell biology. The up-regulation of pro-angiogenic factors helps facilitate the formation of new blood vessels, a process known as angiogenesis. This adaptive response is aimed at increasing oxygen and nutrient supply to hypoxic tissues, including tumors. By stimulating endothelial cell proliferation and migration, as well as influencing the behavior of other cells in the microenvironment, the up-regulation of pro-angiogenic pathways supports the development of a functional vascular network within the tumor and contributes to glioma progression and survival [36,37].

### 2.1. LncRNAs in Adult Gliomas

Adult gliomas primarily affect individuals over the age of 18, with the majority occurring in older adults. The most common adult glioma is glioblastoma multiforme, which is an aggressive and malignant tumor [38]. The CNS5 classification encompasses several types of adult gliomas with varying levels of malignancy. Each glioma type in the CNS5 classification has distinct histopathological features and grading criteria, which take into account factors such as nuclear atypia, mitotic activity, microvascular proliferation, and the presence of necrosis [10]. In addition to histological characteristics, the WHO classification also emphasizes the importance of molecular markers for certain glioma types, such as IDH mutation status, 1p/19q codeletion, and MGMT promoter methylation, which can have prognostic and predictive implications. Non-coding RNAs (ncRNAs) have been reported to play significant roles in glioma progression by regulating gene expression, cellular processes, and the tumor microenvironment [26]. LncRNAs can regulate gene expression through diverse mechanisms, including acting as scaffolds for protein complexes, interacting with DNA, RNA, or proteins, and modulating chromatin structure or transcriptional regulation [39]. Several lncRNAs have been implicated in glioma growth and invasion. Some lncRNAs promote glioma cell proliferation, survival, migration, and invasion by interacting with specific signaling pathways, such as the Wnt/β-catenin pathway or the PI3K/AKT pathway [40,41]. LncRNAs were also found to participate in epigenetic modifications, such as DNA methylation and histone modifications, influencing gene expression patterns in gliomas [42].

Different regulation mechanisms exerted by lncRNAs have been reported to influence adult glioma angiogenesis. LncRNAs can act as competing endogenous RNAs (ceRNAs) by sequestering miRNAs, thereby preventing their binding to target mRNAs. This interaction can modulate the expression of target genes and affect glioma progression [43]. Various lncRNAs have been identified as oncogenes or tumor suppressors in adult gliomas. For example, MALAT1, HOTAIR, and H19 have been found to promote glioma cell proliferation, migration, invasion, and angiogenesis, thereby acting as oncogenes [44,45,46]. Conversely, lncRNAs such as MEG3 and GAS5 have shown tumor-suppressive effects by inhibiting glioma cell growth and inducing apoptosis in adults [47]. Some lncRNAs can bind to proteins and modulate their activity, including transcription factors involved in angiogenesis-related gene expression. By recruiting transcription factors or interacting with other proteins, lncRNAs can regulate pathways that contribute to tumor angiogenesis. LncRNAs can also interact with other RNA molecules, such as mRNAs or miRNAs, to influence their stability, translation, or activity. This interaction can indirectly affect angiogenesis-related processes and pathways. Furthermore, lncRNAs can regulate the behavior and function of neighboring cells, including those within the tumor microenvironment. By influencing the communication between tumor cells and surrounding cells, lncRNAs can impact angiogenesis by promoting or inhibiting the formation of new blood vessels [48,49,50]. LncRNAs have essential roles in adult glioma cell proliferation and migration and guide gene regulation during tumor angiogenesis. They can directly regulate the cytoskeleton or influence the cytoskeleton through various signaling pathways during tumor migration [51]. Tang et al. [52] revealed that the long non-coding RNA-activated by transforming growth factor β (*ATB*) promoted the invasion of adults glioma cells through nuclear factor kappa B (NF-κB) and p38 mitogen-activated protein kinase (MAPK) pathways. Other lncRNAs were found to sponge miRNAs [53]. Dysregulated lncRNAs in gliomas have been associated with clinicopathological features, patient survival, and therapeutic response. They hold potential as diagnostic biomarkers and therapeutic targets for glioma treatment in adults. LncRNAs can modulate gene expression through epigenetic mechanisms in adult glioma. They can interact with chromatin-modifying proteins, such as polycomb repressive complexes (PRC1 and PRC2), to regulate the methylation and acetylation status of histones, thereby impacting gene transcription [54]. LncRNA HOTAIR is known to interact with PRC2 to silence tumor suppressor genes and promote adult glioma progression [55]. LncRNAs can regulate key signaling pathways involved in adult glioma pathogenesis, such as the PI3K/AKT, Wnt/β-catenin, and Notch pathways. They can modulate the expression or activity of pathway components, affecting downstream signaling events and promoting glioma cell survival, proliferation, and invasion. LncRNA CCAT2 has been found to activate the Wnt/β-catenin pathway in glioma cells, contributing to tumor growth [56]. While our understanding of the precise functions and mechanisms of lncRNAs in adult glioma is still evolving, the emerging evidence suggests their important roles in glioma pathogenesis and their potential as therapeutic targets and biomarkers. Further research is needed to unravel the intricate interactions and regulatory networks involving lncRNAs in adult gliomas, paving the way for novel therapeutic strategies and improved patient outcomes.

### 2.2. LncRNAs in Pediatric Gliomas

Malignant brain and central nervous system (CNS) tumors account for ~21% of tumors in children and represent the second leading cause of pediatric cancer deaths after leukemia [57,58]. Pediatric glioma, a common central nervous system tumor in children, is classified into low-grade and high-grade categories. Low-grade gliomas refer to benign, slow-growing grade I or II lesions, while high-grade gliomas encompass rapidly progressing tumors such as anaplastic astrocytoma (grade III), glioblastoma, and diffuse intrinsic pontine glioma (grade IV) [59]. These non-coding RNA molecules have been found to be dysregulated in pediatric gliomas and contribute to tumor development and progression. Various lncRNAs have been identified as tumor suppressors or oncogenes in pediatric gliomas. For example, LINC00312 and MEG3 have been found to act as tumor suppressors, inhibiting glioma cell proliferation and inducing apoptosis [60]. On the other hand, lncRNAs such as MALAT1 and HOTAIR have been implicated as oncogenes, promoting glioma cell growth, invasion, and angiogenesis [61]. LncRNAs can modulate gene expression through epigenetic mechanisms in pediatric gliomas. They can interact with chromatin-modifying proteins and affect the methylation and acetylation status of histones, thereby regulating gene transcription. LncRNA H19, for instance, has been shown to play a role in the epigenetic silencing of tumor suppressor genes in pediatric gliomas [62]. LncRNAs can function as ceRNAs (competing endogenous RNAs) by sponging miRNAs and preventing them from binding to their target mRNAs. This interaction can impact the expression of target genes and affect glioma cell behavior. LncRNA HULC, for example, has been found to regulate miRNA-mediated gene expression in pediatric gliomas [63]. LncRNAs participate in the regulation of signaling pathways that are crucial for pediatric glioma pathogenesis. They can interact with key components of pathways, such as the PI3K/AKT and Wnt/β-catenin pathways, influencing downstream signaling events and cellular processes. LncRNA CCAT2 has been shown to activate the Wnt/β-catenin pathway in pediatric gliomas, promoting tumor growth [64]. Dysregulated expression of specific lncRNAs in pediatric gliomas has been associated with tumor grade, prognosis, and treatment response. These lncRNAs also hold promise as diagnostic and prognostic biomarkers for pediatric gliomas, aiding in patient stratification and personalized treatment decisions [65]. Understanding the roles of lncRNAs in pediatric gliomas provides insights into the molecular mechanisms underlying tumor development and may pave the way for the development of novel therapeutic strategies and targeted therapies.

**Figure 2:**
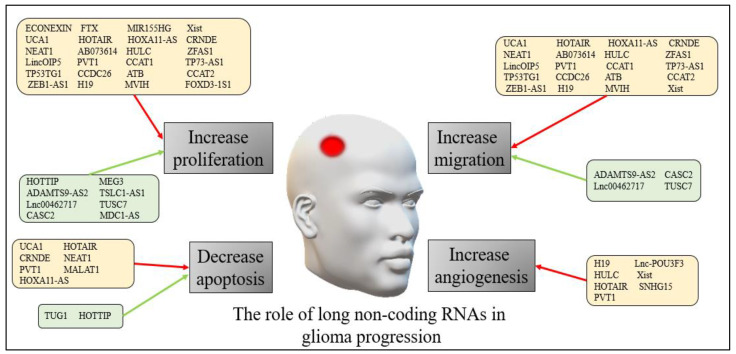
The role of long non-coding RNAs in human glioma progression. The red arrows refer to down-regulation of the non-coding RNAs, while the green arrows refer to up-regulation.

The precise mechanisms through which lncRNAs exert their effects in both adults and pediatric glioma progression are still being explored. However, accumulating evidence suggests that lncRNAs play critical roles in regulating various cellular processes involved in tumor growth, invasion, metastasis, and treatment response. Figure 2 presents a summary of the role of long non-coding RNAs in human glioma progression.

In high-grade gliomas, the high expression of taurine-upregulated gene 1 (*TUG1*) lncRNA has been reported to favor angiogenesis by inhibiting the function of miR-299, which has a binding site for vascular endothelial growth factor (VEGF) [66]. Ye et al. [67] showed that the down-regulation of long intergenic non-protein coding RNA (*LINC01116*) suppresses the angiogenesis of adult glioma in vivo. The same authors suggested that *LINC01116* may play a role in the post-transcriptional regulation of VEGF by binding to miR-31-5p. In the same manner, X-inactive specific transcript (*XIST*) sponges miR-485 in human glioma micro-vascular endothelial cells; hence, the binding of miR-485 enables SRY-box transcription factor 7 (SOX7) to increase the expression level of VEGF [68]. Additionally, Cheng et al. [69] identified a correlation between lncRNA *XIST* and miR-429 in adult glioma cells, which was found to highly affect angiogenesis. The authors also reported that *XIST* knockdown reduced glioma angiogenesis in vitro and in vivo. Likewise, small nucleolar RNA host gene 15 (*SNHG15*) lncRNA knockdown reduced the expression levels of pro-angiogenic factor cell division control protein 42 homolog (CDC42) and VEGF because the miR-153 was longer sponged and led to suppression of tumor angiogenesis in adult glioma cells [70]. RNA binding proteins were found to play critical roles in almost all gene expression steps, especially in transcription as well as post-transcriptional regulations [71]. Yang et al. [72] investigated the expression and the role of lncRNA *LINC00346* in adults glioma angiogenesis regulation and demonstrated that ankyrin repeat and KH domain containing one protein (ANKHD1) and *LINC00346* were highly up-regulated in adult glioma cells, whereas the zinc finger protein 655 (ZNF655) expression was decreased. The authors reported that the inhibition of ANKHD1, *LINC00346,* or the overexpression of ZNF655 impeded angiogenesis of the cells, suggesting that ANKHD1 targeted the long RNA *LINC00346* and enhanced its stability, which then bound to ZNF655 mRNA via their Alu elements. Thereby, *LINC00346* facilitated ZNF655 mRNA degradation via a staufen1 (STAU1) mediated mRNA decay mechanism. Furthermore, the promoter region of ANKHD1 was targeted by ZNF655 and formed the feedback loop of ANKHD1/*LINC00346*/ZNF655 that regulated the angiogenesis of adult glioma cells. Refer to Table 2 for the summary of different lncRNAs mechanisms in adult glioma progression and angiogenesis.

### 2.3. LncRNAs as Biomarkers in Adults and Pediatric Glioma Diagnosis

**Figure 3:**
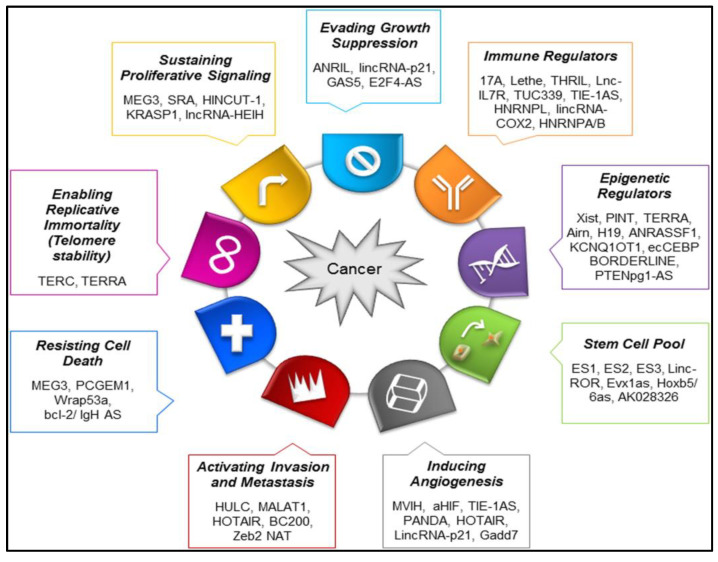
LncRNAs in hallmarks of cancer. Adapted with permission from Parasramka et al. [99].

Long non-coding RNAs (lncRNAs) have shown promise as biomarkers for glioma diagnosis. Their dysregulated expression patterns in gliomas can serve as indicators of disease presence, classification, and prognosis [86]. Dysregulated expression of specific lncRNAs has been observed in gliomas compared to normal brain tissue. This aberrant expression can be detected using various techniques, such as quantitative polymerase chain reaction (qPCR), microarray analysis, or RNA sequencing [87]. The expression levels of certain lncRNAs have demonstrated diagnostic potential for differentiating gliomas from non-neoplastic brain tissue or other brain tumor types. Poor outcome of high-grade glioma patients has been linked to late-stage diagnosis and treatment due to our low knowledge about the early stage of glioma, which cannot be observed with conventional diagnostic approaches [88]. However, even in aggressive therapy of human glioma with a combination of different treatment modalities many types of gliomas have been reported with having a pessimistic prognosis for survival [89,90]. The current gold standard for categorizing nervous system tumors, as outlined in the 2021 edition of the World Health Organization (WHO) classification, prioritizes molecular diagnosis over histologic diagnosis. In many countries, histological evaluation is still in use for different glioma diagnoses, which is difficult and sometimes not clear to acquire tissue because of its special anatomical position in the CNS. Furthermore, diagnosis of glioma using imaging methods such as magnetic resonance imaging and computed tomography before clinical diagnosis or treatment are widely used, but they have failed to improve the early diagnosis rate, which results in significant glioma spreading [91]. Recently, some tumor-related molecules, including different non-coding RNAs, have been reported to be significantly involved in the angiogenesis and progression of gliomas in pediatrics and have been investigated for being diagnostic or prognostic biomarkers [92]. LncRNAs have shown the ability to distinguish between different glioma subtypes, aiding in their accurate classification. For example, lncRNA expression profiles have been used to differentiate between glioblastoma and lower-grade gliomas (LGG), as well as to identify specific molecular subtypes within GBM [93]. The sensitivity, accuracy, and specificity of these molecular biomarkers are highly adequate for the evaluation of early diagnosis or prognosis of human glioma with reliable clinical significance. lncRNAs have multiple biological functions, including transcriptional control, chromatin remodeling, and post-transcriptional processing [94,95]. The expression levels of certain lncRNAs have been associated with the prognosis and overall survival of glioma patients. High expression of some lncRNAs has been correlated with poorer outcomes, including shorter survival times and increased tumor aggressiveness [96]. These lncRNAs can serve as prognostic biomarkers, providing valuable information for patient risk stratification and treatment decision-making. Garcia et al. [97] highlighted the lncRNAs that have been reported with strong evidence for cancer process association and illustrated these lncRNAs in Figure 3 with their influence on the hallmarks of cancer. LncRNAs have the potential to predict the response of gliomas to specific treatments. For example, the expression levels of certain lncRNAs have been associated with resistance to chemotherapy or radiotherapy. Monitoring the expression of these lncRNAs could help identify patients who are more likely to benefit from certain treatment strategies or require alternative therapeutic approaches. Another advantage of lncRNAs as biomarkers is their potential for non-invasive detection. lncRNAs can be detected in various body fluids, including blood, cerebrospinal fluid (CSF), and urine [98]. This enables the development of minimally invasive or liquid biopsy-based approaches for glioma diagnosis and monitoring, reducing the need for invasive procedures, such as surgical biopsies.

Expression abnormalities of lncRNAs and miRNAs have been reported to be associated with the gliomas progression in both adults and pediatrics [100]. Recent studies have indicated that *MALAT1* lncRNA has promising potential to serve as a diagnostic biomarker for gliomas and other cancers with sufficient specificity and sensitivity [26,101]. Up-regulation of the *HOXA-AS3* lncRNA significantly promoted tumor progression, and it can be used to predict poor prognosis in glioma [102]. Wang et al. [103] investigated the role of the *LINC00152* in high-grade glioma in adults and observed its expression was independently associated with poor prognosis of high-grade glioma. The authors also reported that the overall survival of the high-expression glioma group was significantly shorter than the low-expression group, while the knockdown of *LINC00152* inhibited tumor growth in vivo, suggesting *LINC00152* as a potential prognostic biomarker for high-grade glioma, especially in adults. Recent investigations have demonstrated the role of antisense lncRNAs in numerous biological processes, including tumor cell proliferation, invasion, survival, migration, and even apoptosis through multiple epigenetic modifications and chromatin remodeling [104,105]. It was reported that HAND2 antisense RNA 1 (*HAND2-AS1*) in endometrioid endometrial carcinoma was significantly down-regulated, and its overexpression considerably inhibited invasion and metastasis of tumor through inactivating neuromedin U [106]. In a different study, Han et al. [107] found that the *HOXA11-AS* lncRNA could serve as a diagnostic target and biomarker for glioma in adults as well as pediatrics. Similarly, Wu et al. [108] used RNA sequencing data to profile the differentially expressed antisense lncRNAs in various gliomas gathered from the *Chinese Glioma Genome Atlas* database and found convincing evidence of the possibility of using WDFY3 antisense RNA 2 (*WDFY3-AS2*) lncRNA as a novel valuable prognostic biomarker and diagnostic agent for diffuse glioma in adults. Wang et al. [109] analyzed expression profiles of four lncRNA, namely, AGAP2 antisense RNA 1 (*AGAP2-AS1*), long intergenic non-protein coding RNA 1198 (*LINC01198*), TPT1 antisense RNA 1 (*TPT1-AS1*) and MIR155 host gene (*MIR155HG*) of anaplastic astrocytomas patients. The authors found that *TPT1-AS1* was postulated to have a significant protective role, while the other three (*AGAP2-AS1*, *LINC01198,* and *MIR155HG*) were suggested to be involved in tumor development as they were expressed more in the high-risk group.

### 2.4. LncRNAs in Adults and Pediatric Glioma Therapy

**Figure 4:**
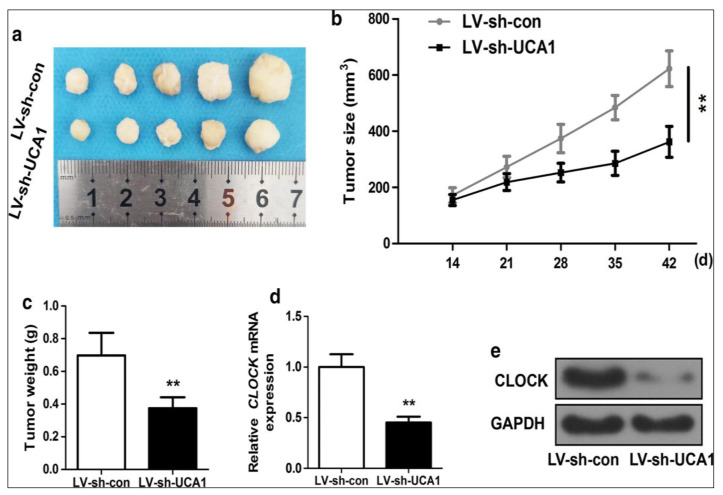
In vivo silencing of UCA1 repressed adult glioma tumor growth. (**a**) In vivo, tumors co-transfected with Lentiviral vector short hairpin control (LV-sh-con) and LV-sh-UCA1. (**b**) The growth curve of glioma tumor. (**c**) Tumor weight. (**d**,**e**) clock circadian regulator protein expression in LV-sh-con and LV-sh-UCA1. ** *p* < 0.01. Adapted from Huang et al. [119] with permission from Springer Nature.

Long non-coding RNAs (lncRNAs) have emerged as potential therapeutic targets in glioma therapy. Their dysregulation in gliomas and involvement in various cellular processes make them attractive candidates for novel treatment strategies. LncRNAs affect cell behavior in several different ways, from epigenetic regulation, including recruitment of transcription factor, splicing regulation, and messenger RNA stability and translation [110]. Owing to the ability of lncRNAs to interact with DNA, RNA, and proteins, lncRNAs also involve chromatin modification, post-transcriptional modifications, and scaffolding [111]. Chen et al. [112] found that up-regulation of the expression of carbamoyl phosphate synthetase 1 intronic transcript 1 (*CPS1-IT1*) lncRNA resulted in a significant decrease in glioma proliferation, migration, and invasion abilities in adults, suggesting *CPS1-IT1* as anti-oncogenic molecules in glioma treatment. In a different study, *lncGRS-1* was found to be a glioma-specific therapeutic target in both adults and pediatrics. Using antisense oligonucleotides that targeted *lncGRS-1,* it was able to selectively decrease tumor growth and sensitize glioma cells to radiotherapy [113]. Ji et al. [114] found that the knockdown of the SWI/SNF complex antagonist associated with prostate cancer 1 (*SChLAP1*) lncRNA suppressed the growth of glioma in adults as it was found to be highly expressed. The authors reported that *SChLAP1* was stabilized by its binding to heterogeneous nuclear ribonucleoprotein L (HNRNPL), which significantly enhanced the interaction with the protein alpha-actinin-4 (ACTN4). Urothelial carcinoma-associated 1 (UCA1) lncRNA was recently discovered as a proto-oncogene in the progression of many cancers, including glioma in adults and pediatrics [115]. UCA1 could promote glioma cell proliferation and cell cycle in adults through the up-regulation of cyclin D1 transcription. Targeting dysregulated lncRNAs through their silencing or inhibition is a potential therapeutic approach. This can be achieved using antisense oligonucleotides (ASOs), small interfering RNAs (siRNAs), short hairpin RNAs (shRNAs), or CRISPR/Cas9-mediated gene editing [116,117,118]. By reducing the expression or function of oncogenic lncRNAs, these strategies aim to inhibit glioma growth, invasion, and other malignant characteristics. Some lncRNAs act as key regulators of gene expression in gliomas. Targeting these lncRNAs can modulate the expression of downstream genes involved in glioma progression. By manipulating the expression or activity of specific lncRNAs, it is possible to influence critical pathways associated with tumor growth, angiogenesis, invasion, and therapy resistance. Huang et al. [119] up-regulated UCA1 in adult glioma cell lines and tissues using lentiviral vector transfections and observed a significant elevation in glioma cell invasion through the induction of epithelial-mesenchymal transition. The same authors reported that the growth of adult glioma cells was significantly suppressed upon the silencing of UCA1 in vivo (Figure 4), and the expression of circadian locomotor output cycles kaput (CLOCK) was also significantly lowered after UCA1 knockdown, suggesting the utility of targeting this lncRNA for adult glioma treatment.

Certain lncRNAs have been reported to participate in epigenetic regulation, influencing chromatin structure and gene expression patterns in gliomas [120]. Targeting these lncRNAs can alter the epigenetic landscape and restore normal gene expression profiles. This approach holds promise for reversing aberrant gene silencing and promoting tumor-suppressive pathways in glioma cells. Combining lncRNA-targeted therapies with other treatment modalities, such as chemotherapy or radiotherapy, is being explored. By targeting lncRNAs alongside traditional treatments, it may be possible to enhance treatment efficacy, overcome therapy resistance, and improve patient outcomes. *LncRNA H19* has been reported in numerous publications as well as its role in promoting cancer cell growth and proliferation [121,122] by regulating the proteins that are involved in apoptosis, cell cycle regulation as well as glucose metabolism, and chemoresistance [123]. *H19* is expressed in both hepatocellular carcinoma and adult glioma-associated endothelial cells within the exosomes, which promote tumor angiogenesis [124]. Zhao et al. [125] observed that the overexpression of *H19* significantly promoted glioma cell proliferation and migration in adults. The autophagy of the tested cell lines was enhanced after the silencing of *H19* and suppressed when the lncRNA was overexpressed. *H19* overexpression inhibited the mechanistic target of rapamycin (mTOR) protein phosphorylation and promoted Unc-51-like kinase 1 (ULK1) phosphorylation. The regulation of mTOR signaling was found to be the mechanism by which *H19* promotes the proliferation, migration, and autophagy of adult glioma cells [125]. In a different study, Sheng et al. [126] recently reported that p53 targeted lncRNA suppressor of tumorigenicity 7 antisense RNA 1 (*ST7-AS1*) acted as a significant tumor suppressor by interacting with polypyrimidine tract-binding protein 1 (PTBP1) to suppress the Wnt/β-catenin signaling pathway in adult and pediatric glioma. They contributed to significant inhibition of glioma cells migration, invasion, and proliferation, in addition to promoting the apoptosis of glioma cells. Down-regulation of PTBP1 occurred as a result of *ST7-AS1* direct binding at the post-transcriptional level. Certain lncRNAs have been implicated in conferring resistance to therapies in gliomas [127]. Targeting these lncRNAs can sensitize glioma cells to standard treatments, making them more responsive to chemotherapy or radiation. This approach aims to enhance the effectiveness of existing therapies and improve treatment responses. In addition to targeting lncRNAs, certain lncRNAs themselves can be used as therapeutic agents. For example, engineered lncRNAs can be designed to exert specific functions, such as acting as decoys for miRNAs or as competitors for protein-protein interactions. These engineered lncRNAs can potentially modulate critical pathways and disrupt glioma progression. Table 3 provides a summary of studies that involved the manipulation of lncRNAs activity for glioma therapy.

## 3. Micro RNAs in Gliomas

Emerging evidence has indicated the significant roles of miRNAs in glioma progression as one of the main factors that regulate the transcription and translation of oncogenes [138]. Micro RNAs have been described as small single-stranded segments (18–25 nucleotide) of ribonucleic acid that play significant roles in different biological processes such as stem cell differentiation and development [139]. In both adults and pediatric glioma progression, miRNAs can exhibit altered expression levels compared to normal brain tissue. Specific miRNAs can act as tumor suppressors or oncogenes, depending on their target genes and functions [140]. miRNAs can act as either oncogenes or tumor suppressors depending on their target genes and functions [141]. Dysregulated expression of specific miRNAs in gliomas can contribute to tumor progression. Oncogenic miRNAs promote glioma cell proliferation, invasion, and angiogenesis by targeting tumor suppressor genes or inhibiting pathways involved in apoptosis. Tumor suppressor miRNAs, on the other hand, inhibit glioma growth and invasion by targeting oncogenes or genes involved in cell cycle regulation and signaling pathways. miRNAs regulate gene expression by binding to the 3’ untranslated region (UTR) of target messenger RNAs (mRNAs). This binding leads to the degradation of target mRNAs or the inhibition of their translation [142]. Dysregulated miRNAs in gliomas can target genes involved in critical cellular processes, including cell cycle control, apoptosis, DNA repair, cell adhesion, and migration. Gliomas require an adequate blood supply for their growth and progression. miRNAs play a crucial role in regulating angiogenesis, the process by which new blood vessels form. Dysregulated miRNAs can target genes involved in angiogenic signaling pathways, such as VEGF and its receptors, modulating the formation of new blood vessels within the tumor microenvironment. Gliomas have the ability to infiltrate and invade adjacent brain tissues, making complete surgical removal difficult. miRNAs are involved in the regulation of processes associated with glioma invasions, such as cell migration, extracellular matrix remodeling, and epithelial-mesenchymal transition (EMT). Dysregulated miRNAs can promote glioma invasion by targeting genes involved in these processes, leading to increased tumor cell motility and invasiveness [143].

### 3.1. Micro RNAs in Adults Gliomas

MicroRNAs were found to influence various aspects of tumor development, progression, and treatment response. In adult gliomas, several miRNAs act as tumor suppressors, inhibiting tumor growth and promoting cell differentiation and apoptosis; miR-124, miR-7, and miR-34a were found to act as tumor suppressors [144]. Conversely, certain miRNAs function as oncogenes, promoting glioma cell proliferation, invasion, and resistance to therapy. MiR-21, miR-10b, and miR-221/222 are among the miRNAs with oncogenic roles in adult gliomas [145]. Deng et al. [146] found that miR-375 showed significantly lower expression in adult glioma cells, while solute carrier family 31 member 1 (SLC31A1) exhibited higher expression. The authors restored the expression of miR-375 and observed significant suppression in glioma cells proliferation, migration, and invasion, as well as the increase in the rate of adult glioma cell apoptosis by targeting SLC31A1. Dysregulated miRNAs can target genes involved in various processes, including cell proliferation, invasion, angiogenesis, and apoptosis. Xia et al. [147] studied the function of miR-15b in human glioma carcinogenesis and reported that its overexpression resulted in cell cycle arrest between G0 and G1 phases of the cell cycle, while suppression of the miR-15b expression resulted in a significant decrease in cell populations in G0/G1 and a corresponding increase in S phase cell populations, suggesting that miR-15b regulates the progression of glioma cells by targeting cell cycle molecules. Epithelial-Mesenchymal Transition (EMT) is a process in which cancer cells acquire mesenchymal characteristics, enabling invasion and metastasis. MiRNAs can regulate EMT in adult gliomas by targeting genes involved in EMT signaling pathways. MiR-200 family members, such as miR-200c and miR-141, are known to inhibit EMT and suppress glioma invasion [148]. Another micro RNA (miR-204-5p) was found to suppress the proliferation of cancer cells. Restoring the expression of miR-204-5p in glioma cells significantly suppressed tumorigenesis and increased overall patient survival [149]. Lin et al. [150] reported that miR-4476 promoted the proliferation, migration, and invasion of adult glioma cells. The mechanistic analyses indicated that the adenomatous polyposis coli (*APC*) was directly targeted by miR-4476 and mediated the oncogenic effects in glioma cells. The overexpression of miR-4476 was found to be an unfavorable prognostic factor and was correlated with the expression of the *c-Jun* proto-oncogene, but negatively correlated with the expression of *APC*, concluding that miR-4476 works as a tumor enhancer, which can stimulate its own expression and furtherly promote the malignant phenotypes of gliomas [150]. MiRNAs play a critical role in regulating angiogenesis, the formation of new blood vessels, which is crucial for tumor growth and progression. MiR-296, miR-126, and miR-210 are examples of miRNAs involved in angiogenesis regulation in gliomas, affecting endothelial cell behavior and angiogenic signaling pathways. MicroRNA genes are directly involved in cancer due to their proximity to fragile sites near many oncogenes. A single miRNA can target multiple mRNAs, and the regulation does not require high complementarity between the miRNA and mRNA. As a result, a single miRNA can specifically regulate the translation of different mRNAs, thereby influencing the synthesis of various proteins [151].

### 3.2. Micro RNAs in Pediatric Gliomas

Micro RNAs have been extensively studied in pediatric gliomas, providing valuable insights into their roles in tumor development, progression, and treatment response. Several miRNAs act as tumor suppressors in pediatric gliomas, inhibiting tumor growth and promoting cell differentiation and apoptosis. MiR-34a, miR-124, and let-7 family members are examples of tumor suppressor miRNAs that are frequently downregulated in pediatric gliomas [152]. Conversely, certain miRNAs function as oncogenes, promoting glioma cell proliferation and invasion. MiR-21 and miR-10b are among the miRNAs with oncogenic roles in pediatric gliomas. The physical properties of miRNAs, which make them resistant to degradation in body tissues and fluids, make them ideal candidates to explore as biomarkers of pediatric malignancies [153]. MiRNA biomarkers are of particular relevance for pediatric malignancies, which typically have a low mutational burden compared with their adult counterparts; thus, the identification of DNA mutations and ctDNA as biomarkers are of more limited use [152]. Neuroblastoma is responsible for approximately 15% of childhood cancer-related deaths and displays clinical variability [154]. Favorable cases may exhibit spontaneous regression, while unfavorable neuroblastoma often leads to high mortality despite intensive treatment. Unfavorable neuroblastoma is commonly associated with MYCN amplification and specific chromosomal abnormalities. Research has shown that MYCN amplification stimulates the upregulation of the miR-17–92 cluster by directly binding to its promoter [155]. The overexpression of the host gene MIRHG1, which is part of the miR-17–92 cluster, has been linked to poor prognosis, higher disease stages, and MYCN amplification [156]. The miR-17–92 cluster is implicated in promoting cell growth in unfavorable neuroblastoma [152,157]. Previous studies have identified a signature of 25 miRNAs that predicts high-risk neuroblastoma patients with a worse prognosis. Interestingly, almost all miRNAs from the miR-17–92 cluster were included in this prognostic signature, and 16 of the 25 miRNAs were regulated by MYC/MYCN [158]. MiRNAs are involved in regulating angiogenesis, a critical process in tumor growth and progression. They can modulate the expression of genes involved in angiogenic pathways, influencing endothelial cell behavior and vessel formation. MiR-296, miR-126, and miR-210 have been implicated in angiogenesis regulation in pediatric gliomas [159]. The dysregulated expression patterns and functional roles of miRNAs in pediatric glioma development, progression, and treatment response highlight their potential as diagnostic biomarkers, prognostic indicators, therapeutic targets, and tools for overcoming treatment resistance.

### 3.3. miRNAs as Biomarkers in Adults and Pediatric Glioma Diagnosis

**Figure 5:**
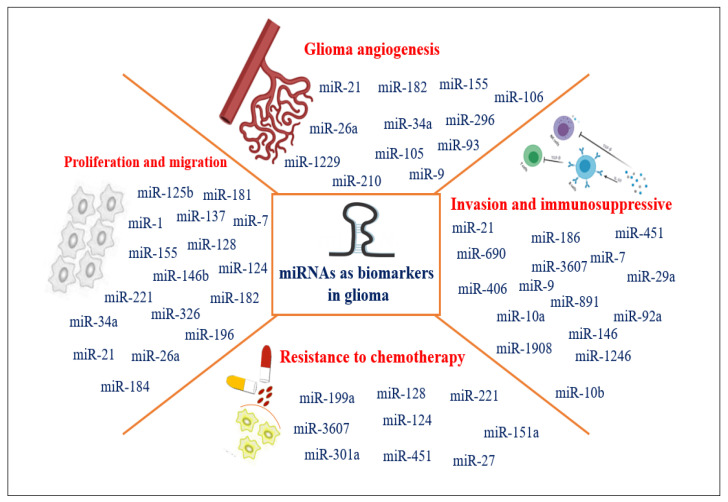
Illustration of miRNAs that play important roles in adults and pediatric glioma regulation as potential biomarkers.

Micro RNAs have emerged as valuable biomarkers for adults and pediatric glioma diagnosis due to their stable expression patterns, tissue specificity, and detectability in various body fluids. Their dysregulation in gliomas compared to normal brain tissue makes them promising candidates for diagnostic purposes. A broad range of miRNAs has been detected in adults and pediatric glioma-derived exosomes, which exert significant functions in the proliferation and invasion of glioma cells through regulating inter-cellular communication in local and distant tumor microenvironments (Figure 5). Many of these miRNAs have been used as biomarkers. Specific miRNAs exhibit dysregulated expression patterns in gliomas compared to normal brain tissue. This dysregulation can serve as a diagnostic indicator, distinguishing gliomas from non-neoplastic brain tissue or other brain tumor types. The expression levels of certain miRNAs can also vary across different glioma subtypes, providing additional molecular information for accurate diagnosis and classification. Yang et al. [160] found that the expression level of miR-221 was significantly increased in high-grade glioma tissue compared to healthy people. The authors also found the serum exosomal miR-221 level significantly higher in glioma patients than it is in controls. The authors concluded that the expression level of exosomal miR-221 in serum increased with the progression of glioma as well as its grades.

Different brain regions and cell types have distinct miRNA expression profiles. By analyzing miRNA expression patterns in glioma tissue samples, it is possible to identify glioma-specific miRNAs that can differentiate between normal brain tissue and gliomas. This tissue-specific expression provides a basis for the development of glioma-specific diagnostic tests. The aberrant expression of miRNAs in tumors has been used as a biomarker for the diagnosis and identification of the level of malignancy of different gliomas [161]. Moreover, circulating miRNAs have been confirmed to be easily detectable in various clinical specimens, such as serum or plasma, with significant high stability, demonstrating great potential for these RNAs as non-invasive and convenient biomarkers [162,163]. miRNAs can be detected not only in tumor tissue but also in various body fluids, including blood, cerebrospinal fluid (CSF), urine, and saliva. This allows for non-invasive or minimally invasive diagnostic approaches, such as liquid biopsy. Analysis of circulating miRNAs in blood or CSF can provide valuable information about glioma presence, progression, and treatment response. The circulating level of miR-182 was found to be significantly higher in glioma patients than that in healthy individuals. Moreover, Xiao et al. [92] observed that high-grade glioma recorded a higher predictive value of circulating miR-182 than the low-grade glioma, suggesting great potential of circulating miR-182 as a noninvasive biomarker for adults and pediatric glioma diagnosis and prognosis. miR21 is another type of miRNA associated with glioma grade. Some studies revealed that miR21 is up-regulated in both low- and high-grade gliomas, while other studies suggested that it is associated with tumor progression and is up-regulated more frequently in high-grade gliomas [164,165,166]. Berthois et al. [167] showed that miR21 is up-regulated in grade IV glioma compared to grade II–III, suggesting that the expression level of miR21 is significantly correlated with the grade of human glioma, and it can be considered as a potential biomarker for tumor progression. miR-137 was found to be aberrantly down-regulated in adult glioma and may act as a tumor suppressor. Thus, the circulatory level of miR-137 has been reported to be associated with clinical features as well as prognosis among adult glioma patients [168]. In a different study, miR-193b was found to be a potential independent prognostic factor for adult glioma. Its overexpression led to an increase in the proliferation, migration, and invasion of adult and pediatric glioma cells, whereas the inhibition of this miRNA resulted in the opposite effects on adults and pediatric glioma behavior [169]. Thus, the overexpression of miR-193b can serve as a useful and accurate biomarker for the diagnosis as well as prediction of prognosis in glioma. Utilizing miRNAs as biomarkers in glioma diagnosis holds promise for improving the accuracy, efficiency, and non-invasiveness of diagnostic approaches. However, further research, standardization, and validation in large patient cohorts are necessary to establish the clinical utility of miRNAs as diagnostic biomarkers in glioma.

### 3.4. Micro RNAs in Adults and Pediatric Glioma Therapy

**Figure 6:**
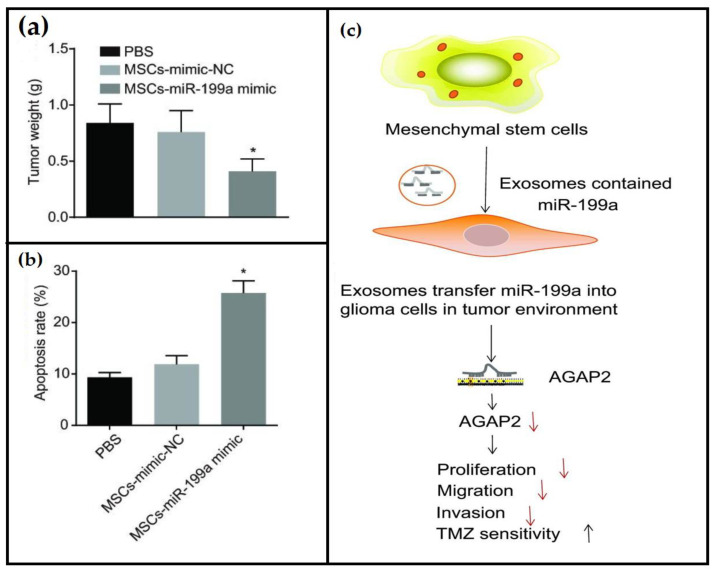
Inhibition of adult glioma cells by using mesenchymal stem cells (MSCs) derived exosomes to deliver miR-199a to glioma cells. (**a**) Tumor weight, (**b**) apoptosis rate of glioma cells, and (**c**) the principal of the approach. PBS—phosphate-buffered saline. * *p* < 0.05. Up and down arrows refer to increasing (↑) or decreasing (↓) the process. Adapted from Yu et al. [174].

Accumulating evidence has revealed abnormal aberrant expression of miRNAs in different types of tumors, which depends on the level of malignancy and/or tumor. Ma et al. [170] performed a meta-analysis for using miRNAs in adults and pediatric glioma diagnosis and revealed that circulating miRNAs are significantly capable of distinguishing human glioma from healthy controls. Considering the circulating miRNAs as a promising diagnostic biomarker for human glioma, which can be used for early detection, Cai et al. [171] demonstrated an inverse association during adult glioma progression between miR-124-3p and RAS homology growth-related (RHOG) expression levels progression in glioblastoma multiforme tissues and cells. The authors revealed that miR-124-3p interacted with RHOG at the RHOG 3’ untranslated region, which inhibits its expression in adult glioma cells. Enrichment of the miR-124-3p expression repressed the transcription of RHOG and thus suppressed adult glioma cell proliferation and promoted their apoptosis. MiR-326 belongs to neuronal micro RNAs, which regulate the expression of numerous neuronal genes in the cortex and cerebellum, and thus, it shows a glioma tumor suppressive role in adults [172]. Sun et al. [173] demonstrated that the expression of miR-19 positively correlates with the grade of glioma. The down-regulation of miR-19 expression led to significant proliferation and invasion inhibition of glioma cells in both adult and pediatric, in addition to the induction of cell cycle arrest (G1) and apoptosis through transcriptional control of RUNX family transcription factor 3 (RUNX3) by binding to its mRNA 3′-UTR as well as repression of transcription activity of the β-catenin/transcription factor 4 (TCF4) in tumor cells. Yu et al. [174] used a different approach for adult glioma treatment based on genetically engineered mesenchymal stem cells for delivering miR-199a (Figure 6). The authors were able to modify the mesenchymal stem cells to release exosomes containing miR-199a, which is known for preventing the development of adult glioma by down-regulating Arf GTPase-activating protein (ArfGAP) with ArfGAP with GTPase domain, ankyrin repeat and PH Domain 2 (AGAP2) MiR-199a was poorly expressed in the glioma tissues and cells while AGAP2 was found to be highly expressed, suggesting that mesenchymal stem cells successfully delivered *miR-199a* to the tumor cells via the released exosomes, resulting in suppression of glioma cells in adults. The same authors reported that the over-expression of miR-199a released from the mesenchymal stem cells significantly enhanced the chemo-sensitivity to the anti-cancer drug temozolomide and inhibited adult glioma cells growth in vivo.

A different study using bioinformatics analysis reported that the peroxisome proliferator-activated receptor alpha (PPARα) acts as a target for miR-19a, among the worse outcomes in clinical glioma patients. The study found a low PPARα expression level, suggesting that PPARα was down-regulated in those patients by the E2F transcription factor 1 (E2F1)/miR-19a signaling [175]. The expression of miR-19a was found to be significantly higher in patients with high-grade glioma compared to those with low-grade. However, the down-regulation of the miR-19a has been linked with the suppression of the proliferation, invasion, and aerobic glycolysis of glioma cells in both adults and pediatric [175]. The expressions of protein tyrosine phosphatase 1B (PTP1B) in glioma tissues and cells were investigated in the study of Shu et al. [176]. They found that the mRNA and protein levels of PTP1B in glioma tissues in adults were higher than those in paired non-tumor tissues. The authors revealed from their TargetScan assay that miR-34c negatively regulated PTP1B and then participated in proliferation regulation. A massive number of publications about different types of micro RNAs provide a promising potential for adult glioma treatment. Table 4 presents a summary of studies that investigated the role of micro RNAs in gliomas.

## 4. Long and Micro RNAs Interaction

**Figure 7:**
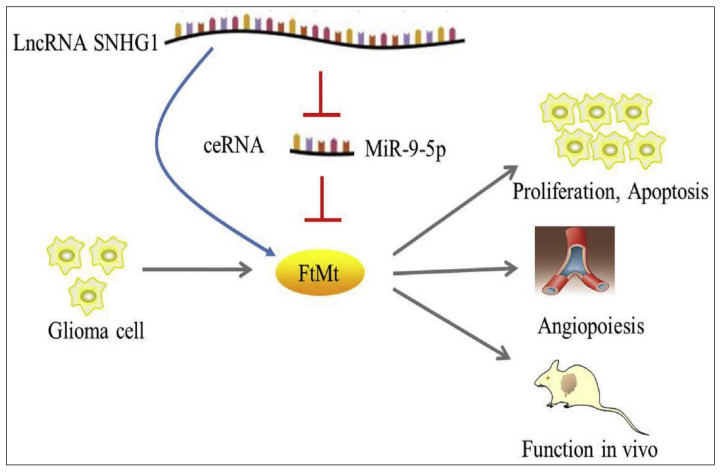
Schematic illustration of the lncRNA SNHG1, miR-9-5p, and mitochondrial ferritin (FtMt) in the promotion of glioma proliferation and angiogenesis. Adapted from Mi et al. [200].

Another significant aspect influencing tumor angiogenesis is the interaction between lncRNAs and miRNAs, which has been reported to affect their respective activities. Recent investigations have revealed that the binding of miRNAs to lncRNAs can lead to the degradation of both molecules [48,196]. Other researchers reported that lncRNAs could act as molecular sponges, which repress the function of the miRNAs, while others suggested a competition between lncRNAs and miRNAs for targeting the same mRNAs [197,198]. Metastasis-associated lung adenocarcinoma transcript 1 (*MALAT-1*) lncRNA was found to sponge miRNAs, which led to a decrease in miR-145 and miR-200, and as a result, the up-regulation of *SOX2* for the SRY-Box protein, increasing cancer stem cell properties. In different studies, miR-9-5p was reported to play a vital role in neurodegenerative disease progression as well as in glioma suppression in adults [199]. Mi et al. [200] investigated the interaction between mitochondrial ferritin (FTMT), miR-9-5p, and *SNHG1* lncRNA in glioma and its impact on adult glioma tumorigenesis and angiogenesis. The authors reported that FTMT was highly expressed in glioma tissues in adults, suggesting its possible role in the progression of glioma in adults and pediatrics. FtMt was also found to be negatively correlated with miR-9-5p while positively related to lncRNA *SNHG1*. However, both *SNHG1* lncRNA and FTMT were found to competitively bind with miR-9-5p (Figure 7) [200]. Currently, numerous potential diagnostic and therapeutic investigations are being developed based on the interactions between the long and micro RNAs, inspired by the significant number of publications that revealed their vital role in glioma proliferation and angiogenesis in both human and mouse glioma.

Metastasis-associated lung adenocarcinoma transcript 1 (*MALAT-1*) lncRNA was found to be targeted by miR-9 for decay inside the cell’s nucleus in glioblastoma cells. miR-9 was also reported to affect tumor angiogenesis by targeting VEGF [201]. *TUG1* lncRNA was reported to accelerate glioblastoma angiogenesis by the up-regulation of VEGF gene expression through the binding to miR-299 [197]. The long intergenic noncoding RNA p21 (*lincRNA-p21*) was found to be activated by the tumor protein TPP53 and to was shown to bind to let-7 miRNA, which led to angiogenic functions inhibition [202]. On the contrary, Yoon et al. [203] found that the overexpression of let-7 miRNAs led to a degradation of *lincRNA-p21*. In a different study, Yu et al. [204] reported that *TUG1* lncRNA may act either as an oncogenic lncRNA or tumor suppressor. The extended regulation of tumor angiogenesis by the interaction between lncRNAs and miRNAs offers a relatively new and promising research field with great possibilities for therapeutic discoveries.

Studying the interaction between lncRNAs and miRNAs in gliomas and glioblastomas is important for several reasons. lncRNAs and miRNAs are key components of gene regulatory networks. Understanding their interplay can provide insights into the complex regulatory mechanisms involved in glioma and glioblastoma development and progression. This knowledge can uncover new potential therapeutic targets or diagnostic markers. Both lncRNAs and miRNAs have been shown to play critical roles in modulating gene expression. Dysregulation of gene expression is a hallmark of gliomas and glioblastomas. Investigating how lncRNAs and miRNAs interact, and influence gene expression can shed light on the molecular mechanisms underlying these diseases. Identifying specific lncRNA-miRNA interactions that are dysregulated in gliomas and glioblastomas can potentially serve as diagnostic or prognostic biomarkers. Additionally, understanding these interactions may reveal potential therapeutic targets for developing novel treatments or personalized therapies for these aggressive brain tumors. The interaction between lncRNAs and miRNAs can influence various processes related to tumor growth, invasion, angiogenesis, and response to therapy. Elucidating these interactions can provide valuable information on disease progression and patient prognosis, aiding in clinical decision-making. For example, recent investigations reported abnormal high expression of the *SNHG1* lncRNA in glioma cells, suggesting its potential role in glioma progression and poor prognosis of tumors [197,205]. *SNHG1* has been reported to potentiate the progression of human glioma through sponging miR-194 to regulate PHLDA1 expression [206]. Finally, manipulating the interaction between lncRNAs and miRNAs holds promise as a therapeutic strategy. Targeting specific lncRNA-miRNA interactions may allow for the modulation of critical signaling pathways involved in glioma and glioblastoma development, offering new avenues for treatment.

**Figure 8:**
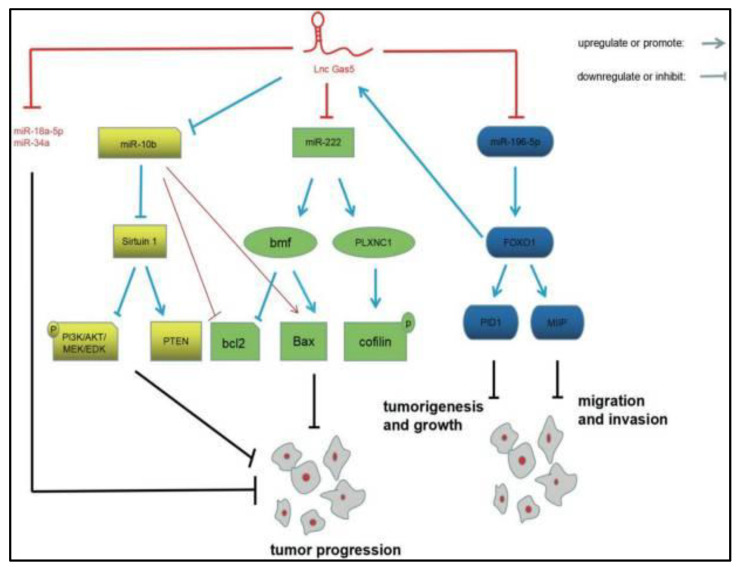
Illustration of the interactions between lncRNA GAS5 and the other miRNAs in human glioma pathogenesis. Adapted from Tan et al. [212].

At a glance, lncRNAs have been reported to act as RNA sponges, competing endogenous RNAs, which can specifically bind to other non-coding RNAs (miRNAs) at a specific sequence. This binding prevents miRNAs to target specific mRNAs (competitive inhibition) by decreasing the available amount of free miRNA [138,207]. These interactions stimulate different pathways, such as the lncRNAs-miRNAs-NF-κB molecular signaling pathway [208], the lncRNAs-miRNAs-Wnt-β-catenin molecular signaling pathway [209], and the lncRNAs-miRNAs-MAPK kinase molecular signaling pathway [210]. Ding et al. [211] recently discovered that growth arrest-specific 5 (GAS5) could repress miR-10b expression in human gliomas to further suppress tumor progression. The authors reported that *GAS5* significantly down-regulated miR-10b and also reduced the expression of sirtuin 1 (SIRT1), thereby hindering the phosphorylation of each of protein kinase B (AKT), mitogen-activated protein kinase (MEK), phosphoinositide 3-kinase (PI3K) and extracellular signal-regulated kinase (ERK), leading to increase the expression of phosphatase and tensin homolog (*PTEN*) gene and inhibit glioma proliferation. Tan et al. [212] studied the roles of lncRNA *GAS5* in glioma progression and its interaction with the other miRNAs (Figure 8). The authors reported that GAS5 inhibits the expression of various miRNAs in glioma development, including miR-222, miR-10b, miR-18a-5p, miR-196-5P, and miR-34a. It has also been reported that these interactions are involved in the glioma pathogenesis through numerous mechanisms, including those mediated by Bcl-2-modifying factor (BMF), Forkhead box protein O1 (FOXO1), B-cell lymphoma 2 (BCL2), Bcl-2-associated X protein (BAX), plexin-C1 (PLXNC1), cofilin, phosphotyrosine interaction domain-containing protein 1 (PID1), migration and invasion inhibitory protein (MIIP), SIRT1, PI3K, AKT, MEK, ERK, and PTEN.

A recent investigation revealed that lncRNAs mostly exert their function through specific binding to RNA-binding proteins, which can also interact with other RNAs besides the lncRNAs [213]. Various proteins that regulate molecular functions of normal and glioma cells can bind with lncRNAs. Heterochromatin protein 1 (HP1) and DNA methyl transferases (DNMT) are two regulatory proteins classified as RNA-binding proteins. LncRNA *MALAT-1* has been reported to interact with a family of proteins known as SR proteins to regulate the splicing of many genes, while the lncRNA *HOTAIR* interacts with lysine-specific demethylase 1 (LSD1) and polycomb repressive complex 2 (PRC2) to regulate the epigenetic status of numerous oncogenes [214]. Chen et al. [215] investigated the function and mechanism of the HLA complex group 11 (*HCG11*) lncRNA in human glioma and observed a low level of *HCG11* expression in glioma samples compared with the sample of healthy people. The authors reported that the binding of FOXP1 protein to *HCG11* led to its transcriptional inactivation, which was caused by its low levels. The same authors suggested that *HCG11* acted as a competing endogenous RNA in glioma cells by sponging micro-496 to up-regulate cytoplasmic poly-adenylation element binding protein 3, which, together with miR-496 is involved in the progression of glioma. Table 5 presents the summary of studies on the interactions between lncRNAs and miRNAs in gliomas.

## 5. Future Directions and Clinical Relevance

Future research efforts should focus on unraveling the precise functional mechanisms of lncRNAs and miRNAs in glioma progression. Understanding how these non-coding RNAs interact with specific target genes and signaling pathways will provide crucial insights into their roles and therapeutic potential. The identification and validation of lncRNAs and miRNAs as diagnostic, prognostic, and predictive biomarkers in gliomas should be further explored. Developing comprehensive panels of lncRNAs and miRNAs that can improve accuracy in diagnosis, risk stratification, and treatment selection would be valuable for personalized medicine. Investigating the therapeutic potential of lncRNAs and miRNAs in glioma therapy is an important direction. Targeting dysregulated lncRNAs and miRNAs with novel therapeutic approaches, such as antisense oligonucleotides, small molecule inhibitors, or gene-editing technologies, could provide new treatment options for glioma patients. Considering the interplay between lncRNAs, miRNAs, and other molecular alterations in gliomas, future studies should explore combination therapies targeting multiple dysregulated pathways. Combining lncRNA and miRNA-based therapies with existing treatment modalities, such as chemotherapy, radiation, or targeted therapies, may enhance treatment efficacy and overcome resistance.

The analysis of lncRNAs and miRNAs in gliomas holds clinical relevance in several aspects, including the diagnostic and prognostic biomarkers. Identification and validation of lncRNAs and miRNAs as biomarkers can aid in glioma diagnosis, risk stratification, and predicting patient outcomes. They can provide valuable information for personalized treatment decisions. Dysregulated lncRNAs and miRNAs can serve as potential therapeutic targets. Developing targeted therapies against these non-coding RNAs may improve treatment outcomes and overcome therapy resistance. The detection of lncRNAs and miRNAs in body fluids offers non-invasive or minimally invasive diagnostic opportunities, such as liquid biopsy. This could revolutionize glioma diagnosis and monitoring, allowing for more frequent assessments and treatment adjustments. lncRNAs and miRNAs contribute to molecular subtyping and classification of gliomas. By analyzing their expression profiles, distinct glioma subtypes can be identified, which have varying clinical characteristics, treatment responses, and prognoses. Molecular subtyping based on lncRNAs and miRNAs can aid in personalized treatment strategies and guide clinical decision-making. Dysregulated expression of specific lncRNAs and miRNAs has been associated with treatment response and resistance in gliomas. Their expression profiles can potentially predict the efficacy of different treatment modalities, including chemotherapy, radiation therapy, and targeted therapies. Integrating lncRNA and miRNA data into treatment planning may help optimize therapeutic strategies and improve patient outcomes. lncRNAs and miRNAs are emerging as potential therapeutic targets in glioma. Modulating their expression or activity holds promise for developing novel treatment strategies. Targeting specific lncRNAs or miRNAs involved in glioma progression can potentially influence critical pathways, enhance treatment responses, and overcome therapy resistance. The detection of lncRNAs and miRNAs in body fluids, such as blood or cerebrospinal fluid, enables non-invasive monitoring of glioma. Liquid biopsy-based approaches can provide real-time information about tumor dynamics, treatment response, and the emergence of therapy-resistant clones. This allows for regular monitoring of the disease without the need for repeated invasive procedures. The clinical relevance of lncRNAs and miRNAs in glioma is multifaceted. They hold promise as diagnostic and prognostic biomarkers, aid in molecular subtyping, predict treatment response, serve as therapeutic targets, and enable non-invasive monitoring. Incorporating lncRNA and miRNA information into clinical practice has the potential to enhance personalized treatment approaches and improve patient outcomes in glioma.

## 6. Conclusions

Both long non-coding RNAs (lncRNAs) and Micro RNAs (miRNAs) play important roles in glioma progression, and their involvement provides insights into potential future directions and clinical relevance. Dysregulated lncRNAs have been implicated in various aspects of glioma progression, including cell proliferation, invasion, angiogenesis, and therapy resistance. They can act as oncogenes or tumor suppressors by regulating gene expression, chromatin structure, and cellular signaling pathways. Specific lncRNAs have been identified as diagnostic or prognostic biomarkers in gliomas. Targeting dysregulated lncRNAs holds promise for developing novel therapeutic strategies. However, further research is needed to elucidate the functional mechanisms of lncRNAs and validate their clinical relevance. Dysregulated miRNAs in gliomas are involved in the regulation of gene expression, cellular processes, and the tumor microenvironment. They can act as oncogenes or tumor suppressors by targeting specific mRNA transcripts and modulating critical pathways. MiRNAs have implications for glioma diagnosis, prognosis, and treatment response prediction. Their detection in body fluids offers non-invasive diagnostic opportunities. Manipulating miRNA expression or activity represents a potential therapeutic approach. However, further research is required to fully understand the functional roles of miRNAs and translate these findings into clinical practice. Researchers are exploring the therapeutic potential of miRNA-based therapies, such as using synthetic miRNA mimics to restore the expression of tumor-suppressive miRNAs or using antagomirs to inhibit oncogenic miRNAs. LncRNAs, on the other hand, are longer RNA molecules that have diverse regulatory functions, including acting as molecular scaffolds, decoys, or guides for chromatin-modifying complexes. Dysregulation of lncRNAs has been implicated in cancer development and progression, including gliomas. Targeting specific lncRNAs with antisense oligonucleotides or small interfering RNAs (siRNAs) holds promise for therapeutic intervention in cancer. Non-coding RNAs also serve as potential biomarkers for cancer diagnosis, prognosis, and therapeutic response. Their expression patterns can provide valuable information about the molecular subtype of tumors and help guide personalized treatment approaches. Overall, non-coding RNAs offer exciting avenues for cancer therapy, and ongoing research aims to further unravel their mechanisms and develop innovative RNA-based therapeutic strategies. The current state of molecular therapy is still in the developmental stage, as most of the investigations prove the role of a particular molecule in a particular process and suggest potential applications of it. The next few years will witness the end of many incurable malignant diseases, including different gliomas.

## Figures and Tables

**Table 1:** The latest World Health Organization (WHO) classification (CNS5).

No.	Major Category	Type	Grade
1	Diffuse Astrocytic and Oligodendroglial Tumors	Diffuse astrocytoma	(grade II)
		Anaplastic astrocytoma	(grade III)
		Glioblastoma	(grade IV)
2	Oligodendroglial Tumors	Oligodendroglioma	(grade II)
		Anaplastic oligodendroglioma	(grade III)
3	Mixed Oligoastrocytic Tumors	Oligoastrocytoma	(grade II)
		Anaplastic oligoastrocytoma	(grade III)
4	Ependymal Tumors	Subependymoma	(grade I)
		Myxopapillary ependymoma	(grade I)
		Ependymoma	(grade II-III)
		Anaplastic ependymoma	(grade III)
5	Choroid Plexus Tumors	Choroid plexus papilloma	(grade I)
		Atypical choroid plexus papilloma	(grade II)
		Choroid plexus carcinoma	(grade III)
6	Other Rare Gliomas	Pleomorphic xanthoastrocytoma	(grade II)
		Rosette-forming glioneuronal tumor of the fourth ventricle	(grade II)
		Angiocentric glioma	(grade I)
		Papillary glioneuronal tumor	(grade I)
		Dysembryoplastic neuroepithelial tumor	(grade I)

**Table 2:** The effect and mechanism of lncRNAs on glioma progression.

LncRNA	The Effect on Glioma Progression	Ref.
HOTAIR	Cell cycle progression and promotion of adult glioma invasion by targeting enhancer of zeste homolog 2 (EZH2), vascular endothelial growth factor (VEGF), and matrix metalloproteinases (MMP-7 and MMP-9).	[73,74]
SNHG6	Regulate glioma cell proliferation in adults through targeting of P21.	[75]
MALAT1	Promotes progression and proliferation through miR-199a-zinc fingers and homeoboxes protein 1 (ZHX1) regulation and sponging of the miR-101.	[76,77]
LINC00689	Promote the proliferation of adult glioma cells through targeting of miR-338-3p/pyruvate kinase M2 (PKM2) axis.	[78]
GAS5	Reduction of adult glioma proliferation, and invasion and induces apoptosis by sponging miR-18a-5p, miR-222, and miR-424.	[79,80]
TUG1	Promote migration and differentiation of adult glioma cells by functioning as a competing endogenous RNA for miR-6321.	[81]
H-19	Induces proliferation, invasion, and metastasis of adult glioma cells in adults through the regulation of miR-140 and miR-29a.	[82,83]
CCAT2	Enhance angiogenesis of glioma in adults and inhibit endothelial cell apoptosis through the stimulation of VEGFA expression and secretion.	[84]
POU3F3	Promote glioma angiogenesis in adults by up-regulating fibroblast growth factor/receptor β (bFGF/bFGFR) and VEGFA expression.	[85]

**Table 3:** Summary of the commonest LncRNAs that have been used in glioma treatment.

Study	Type of LncRNA	Target	Remark
Wen et al. [128]	*LBX2-AS1*	AKT/GSK3β pathway	Silencing of LBX2 antisense RNA 1 (*LBX2-AS1*) suppressed glioma cells proliferation in adults and pediatric and increased apoptosis by the protein kinase B (AKT)- glycogen synthase kinase 3β (GSK3β) pathway
Qin et al. [129]	*TSLC1-AS1*	B-RAF proto-oncogene (BRAF) oncogene	Tumor suppressor in lung cancer 1 antisense RNA 1 (*TSLC1-AS1*) up-regulation resulted in a significant inhibition in the proliferation of adults and pediatric glioma cells.
Xu et al. [130]	*HOTTIP*	BRE gene	Overexpression of HOXA distal transcript antisense RNA (*HOTTIP*) promoted adult glioma cells apoptosis and inhibited their growth by down-regulating brain and reproductive organ-expressed protein expression.
Li et al. [131]	*SLC26A4-AS1*	NPTX1 and NFKB1	Solute carrier family 26 member 4 antisense RNA 1 (*SLC26A4-AS1*) overexpression promoted neuronal pentraxin 1 (NPTX1) expression by recruiting NFKB1 leading to an anti-angiogenic effect.
Bi et al. [132]	*NEAT1*	let-7g-5p/MAP3K1 axis	Nuclear-Enriched Abundant Transcript 1 (*NEAT1*) down-regulation restrained malignant behaviors of glioma stem cells in adults and pediatric.
Wang et al. [133]	*ENST00462717*	MDM2/MAPK	LncRNA *ENST00462717* suppressed the cell’s activities by inhibiting Mouse double minute 2 homolog (MDM2)/MAPK pathway.
Han et al. [134]	*MALAT1*	MMP2 gene	Metastasis-associated lung adenocarcinoma transcript 1 (*MALAT1*) overexpression suppressed cancer cells proliferation by down-regulation of matrix metalloproteinase 2 (MMP2) gene and extracellular signal-regulated kinase (ERK)/MAPK signaling inactivation.
Li et al. [135]	*TUG1*	gene 1	*TUG1* expression up-regulated gene 1 in glioma tissues in adults and acted as a tumor suppressor by promoting cell apoptosis.
Luo et al. [136]	*MEG3*	HIF1α	Overexpression of maternally expressed gene 3 (*MEG3*) inhibited the proliferation and invasion of tumor cells by suppressing the expression of hypoxia-inducible factor 1α (HIF1α).
Li et al. [137]	*Linc01060*	MZF1/c-Myc	Long intergenic non-protein coding RNA 1060 (*Linc01060*) promoted myeloid zinc finger 1 (MZF1)-mediated c-MYC protooncogene transcriptional activities and thus promoted glioma progression in adults and pediatric.

**Table 4:** Summary of the commonest micro RNAs that have been used in adults and pediatric glioma treatment.

Study	Type of miRNA	Target	Remark
Xi et al. [177]	miR-29a	Quaking gene isoform 6	Up-regulation of miR-29a inhibited the expression of *QKI-6* gene and thus inhibited the malignant behavior of adult glioma.
Jia et al. [178]	miR-19a	Akt protein	miR-19a/b knockdown inhibited glioma cells proliferation and invasion in adult and pediatric by down-regulating Akt expression.
Ma et al. [179]	miR-101	COX-2 enzyme	miR-101 significantly down-regulated the expression of COX-2 leading to inhibition of tumor development.
Rani et al. [180]	miR-145	Sox9 and ADD3 proteins	miR-145 up-regulation has a tumor-suppress the activity of oncogenic proteins Sox9 and ADD3 leading to reduction in glioma activities both in adults and pediatrics.
Du et al. [181]	miR-326	SMO oncogene	miR-326 overexpression repressed SMO oncogene and inhibited adult glioma biological behaviors.
Gong et al. [182]	let-7e	NRAS protein	let-7e up-regulation led to inhibition proliferation and migration of adult glioma cells by reducing the expression of NRAS oncoprotein.
Yang et al. [183]	miR-203a	IFN-stimulated gene	Up-regulation of miR-203a promoted the interferon response, leading to suppression of adult glioma proliferation.
Jiang et al. [184]	miR-23b	PI3K/Akt signaling	miRNA-23b up-regulation suppressed PI3K/Akt signaling pathway leading to inhibition of glioma cells activities.
Xue et al. [185]	miR-182-5p	PCDH8 gene	miR-182-5p negatively regulated the expression of tumor suppressor gene PCDH8, leading to enhance adult glioma progression.
Sun et al. [186]	miR-137	Rac1 gene	Overexpressed miRNA-137 Inhibited the growth of glioma cells by directly targeting Rac1 gene.
Wang et al. [187]	miR-21	SPRY2 gene	miR-21 significantly increased glioma cells resistance to carmustine by decreasing the expression of SPRY2 protein.
Chen et al. [188]	miRNA-107	VEGF	Up-regulation of miR-107 significantly inhibited the angiogenesis of glioma cells and the expression of VEGF.
Qin et al. [189]	miR-142	Rac1 gene	miR-142 inhibited glioma migration and invasion by direct targeting Rac1.
Liu et al. [190]	miR-140	ADAM9 protein	miR-140 up-regulation inhibited glioma cells proliferation and invasion via suppressing the expression of ADAM9 protein.
Lan et al. [191]	miR-144-3p	c-Met protein	Up-regulation of miR-144-3p inhibited survival capability in glioma cells and increased apoptosis by targeting c-Met.
Yan et al. [192]	miR-155	Wnt signaling pathways	miR-155 promoted glioma cells progression by promoting Wnt signaling pathways.
Sun et al. [193]	miR-152-3p	DNMT1 gene	miR-152-3p up-regulation directly knocked down DNMT1 and led to significant induction of glioma cells apoptosis.
Dong et al. [194]	miR-429	SOX2 gene	MiR-429 suppressed the proliferation of human glioma cells by targeting SOX2.
Zhou et al. [195]	miR-181b	Sal-Like Protein 4	miR-181b up-regulation significantly decreased glioma cells proliferation and Invasion in adults via Targeting SALL4.

**Table 5:** Summary of the most common micro RNAs that have been associated with glioma pathophysiology.

Study	lncRNA/miRNAInteraction	Remark
Ma et al. [216]	*ATB*/miR-200a	LncRNA activated by TGF-beta (*ATB*) up-regulation inhibits miR-200a in adult glioma cells and facilitates the transforming growth factor β2 (TGF-β2) signaling, while its knockdown significantly inhibits glioma cell growth/proliferation.
[53]	*BCYRN1*/miR-619-5p	Brain cytoplasmic RNA 1 (*BCYRN1*) acted as competing endogenous RNA, which inhibits glioma progression by sponging miR-619-5p and regulating the expression of the CUE domain-containing protein 2 (*CUEDC2*) gene.
Yang et al. [217]	*NEAT*1/miR-107 miR-7e-5p	This binding prevented the repression of cell division protein kinase 6, resulting in increased cancer cells growth.
Cui et al. [218]	*CCAT1*/miR-181b	lncRNA colon cancer-associated transcript 1 (*CCAT1*) knockdown notably suppressed glioma cell proliferation and promoted apoptosis by acting as a competing endogenous RNA for miR-181b.
Qin et al. [219]	*MEG3*/miR-19a	Suppression of glioma cells proliferation and invasion in adults by acting as a competing endogenous RNA for miR-19a.
Sun et al. [220]	*UCA1*/miR-122	*UCA1* acted as an endogenous sponge for miR-122. The binding led to miR-122 downregulation and significant suppression in all glioma cell activities.
Gong et al. [182]	*NEAT1*/let-7e	*NEAT1* up-regulation reduced let-7e expression, which seemed to suppress tumor function. Thus, *NEAT1* knockdown led to let-7e overexpression and reduced the oncoprotein NRAS expression.
Wang et al. [221]	*CASC2*/miR-21	Cancer susceptibility candidate 2 (CASC2) suppresses glioma cell proliferation via the negative regulation of miR-21.
Shang et al. [222]	*TUSC7*/miR-23b	Tumor suppressor candidate 7 (*TUSC7*) up-regulation suppressed the activities of glioma cells in adult and led to the down-regulation of miR-23b.
Zheng et al. [223]	*CRNDE*/miR-186	Colorectal neoplasia differentially expressed (*CRNDE*) knockdown positively regulated miR-186 expression and suppressed the activity of glioma cells.
Su et al. [224]	*SOX2OT*/miR-122 and miR-194-5p	Knocking down of SOX2 overlapping transcript (*SOX2OT*) inhibited the malignant biological behaviors of glioma cells via miR-122 and miR-194-5p up-regulation.
Ma et al. [225]	*MALAT/*miR-140	Metastasis-associated lung adenocarcinoma transcript 1 (*MALAT1*) downregulation increased the blood–tumor barrier and the permeability by up-regulating miR-140.
Cai et al. [226]	*TUG1*/miR-144	Taurine upregulated gene 1 (*TUG1*) regulated blood-tumor barrier permeability in adult and pediatric glioma by targeting miR-144.
Zhang et al. [227]	*TP73-AS1*/miR-142	TP73 antisense RNA 1 (*TP73-AS1*) promoted the growth of adult glioma via sponging miR-142 and acting as a competing endogenous RNA to promote high mobility group box 1 (HMGB1) expression.
Cui et al. [218]	*CCAT1*/miR-181b	Knockdown of colon cancer-associated transcript 1 (*CCAT1*) inhibited glioma proliferation by sponging miR-181b, resulting in de-repression of its endogenous targets platelet-derived growth factor receptor (PDGFRα) and fibroblast growth factor receptor 3 (FGFR3).
Ma et al. [228]	*PVT1*/miR-186	The overexpression of plasmacytoma variant translocation 1 (PVT1) increased autophagy related 7 (*ATG7*) and *Beclin1* expression by targeting miR-186, thus promoting the proliferation of glioma cells.
Ke et al. [229]	*HOTAIR*/miR-326	HOX transcript antisense intergenic RNA (HOTAIR) knockdown inhibited the malignant behaviors of glioma cells in adults through the modulation of miR-326.
Zheng et al. [230]	*CRNDE*/miR-384	Knockdown of colorectal neoplasia differentially expressed (*CRNDE*) and miR-384 overexpression significantly led to tumor regression.
Liu et al. [231]	*CASC2c*/miR-101	Overexpression of cancer susceptibility 2 (*CASC2c*) promoted the malignant behavior of adult glioma cells by directly bounding with miR-101.
